# A patient tumour-on-a-chip system for personalised investigation of radiotherapy based treatment regimens

**DOI:** 10.1038/s41598-019-42745-2

**Published:** 2019-04-19

**Authors:** R. Kennedy, D. Kuvshinov, A. Sdrolia, E. Kuvshinova, K. Hilton, S. Crank, A. W. Beavis, V. Green, J. Greenman

**Affiliations:** 1 Department of Biomedical Sciences, The University of Hull, Cottingham Road, Hull, UK; 2 School of Engineering & Computer Science, The University of Hull, Cottingham Road, Hull, UK; 3 Department of Medical Physics, Hull and East Yorkshire Hospitals NHS Trust, Cottingham, UK; 4 Department of Chemical & Biological Engineering, The University of Sheffield, Sheffield, UK; 5 Department of Maxillofacial Surgery, Hull and East Yorkshire Hospitals NHS Trust, Hull, UK; 6 Faculty of Health and Well Being, Sheffield-Hallam University, Sheffield, UK

**Keywords:** Experimental models of disease, Head and neck cancer

## Abstract

Development of personalised cancer models to predict response to radiation would benefit patient care; particularly in malignancies where treatment resistance is prevalent. Herein, a robust, easy to use, tumour-on-a-chip platform which maintains precision cut head and neck cancer for the purpose of *ex vivo* irradiation is described. The device utilises sintered discs to separate the biopsy and medium, mimicking *in vivo* microvascular flow and diffusion, maintaining tissue viability for 68 h. Integrity of tissues is demonstrated by the low levels of lactate dehydrogenase release and retained histology, accompanied by assessment of cell viability by trypan blue exclusion and flow cytometry; fluid dynamic modelling validates culture conditions. An irradiation jig is described for reproducible delivery of clinically-relevant doses (5 × 2 Gy) to newly-presenting primary tumours (n = 12); the addition of concurrent cisplatin is also investigated (n = 8) with response analysed by immunohistochemistry. Fractionated irradiation reduced proliferation (BrdU, p = 0.0064), increased DNA damage (ƴH2AX, p = 0.0043) and caspase-dependent apoptosis (caspase-cleaved cytokeratin-18) compared to control; caspase-dependent apoptosis was further increased by concurrent cisplatin compared to control (p = 0.0063). This is a proof of principle study showing the response of cancer tissue to irradiation *ex vivo* in a bespoke system. The novel platform described has the potential to personalise treatment for patients in a cost-effective manner with applicability to any solid tumour.

## Introduction

The requirement for targeted and personalised treatment is increasingly apparent; head and neck squamous cell carcinomas (HNSCC) treated with chemotherapy or radiotherapy have 5-year overall survival figures of only 40–60%^1,2^, with resistance to radiation being a key factor^2^.

No standard-of-care biomarkers are currently employed for HNSCC in the UK^3^. Some centres screen for HPV-positive cancers as this is the strongest predictor of locoregional control and disease specific survival^4–6,^ and phase III treatment de-escalation trials are underway^7^. Aside from these trials, no technologies or screening methodologies, aiming to personalise therapy for HNSCC are currently employed. This could be attributable to the limited ability of pre-clinical models to reliably predict clinical outcome^8^.

Current *in vitro* research models fail to recapitulate the complexities of the *in vivo* 3-dimensional (3D) architecture and environment of a tumour. Cell lines have been widely employed^9^; however increasing evidence highlights the critical nature of the 3D structure^10,11^, which has led to engineered tissues being constructed from layered cell lines^12–14^. Whilst these engineered tissues hold significant benefits over traditional monolayer culture and have been integrated into on-chip systems^15,16^, *in vivo* complexities are still not entirely mirrored, as the full gamut of cell types and extracellular matrix found in primary tissues are absent.

Static culture of primary tumour samples has been widely reported^17,18^ affording more faithful mimicry of *in vivo* characteristics. Further development to include the use of primary samples within on-chip platforms confers additional benefits pertaining to perfusion, with continuous delivery of nutrients, removal of waste products and the ability for repeated effluent sampling^19,20^. The maintenance of primary tissues in microfluidic devices has been successfully shown by ourselves and others over 2–14 days depending on the human tissue type^21–27^. The use of primary tissue on these platforms is paramount as the intricate structure and organisation of cells within tissues is crucial for correct function^28,29^. A major advantage of the current device over those previously described by Zambon^23^ and Atac^25^, who both maintain primary tissue (adipose, and skin and hair respectively) under continuous flow, is that the system is simple and highly reliable, and thus can easily be transferred into a clinical setting.

**Figure 1:**
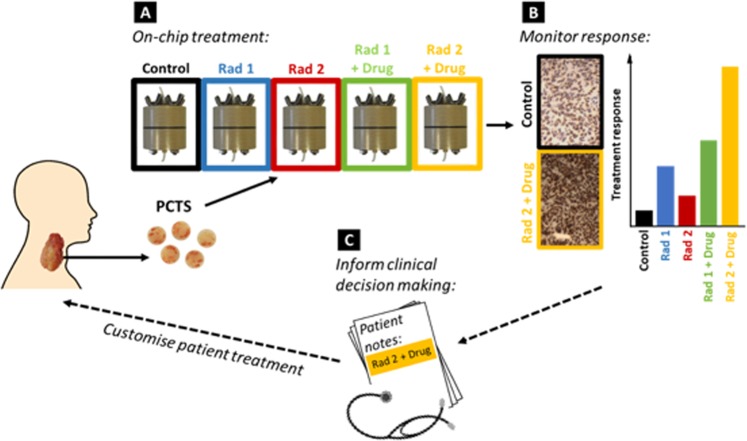
Schematic detailing the interrogation of tumour on-chip to inform patient treatment. Tissue from surgical resection is sliced, generating precision cut tumour slices (PCTS), which are loaded into tumour-on-a-chip devices (**A**) and treated with radiation based regimens. (**B**) After treatment PCTS are analysed using specific markers and compared to controls. (**C**) Results fed back to clinicians could provide information to develop personalised regimens. Solid lines detail methodology employed within the study, dotted lines indicate workflow for development.

Radiation based regimens are a mainstay in the treatment of HNSCC, yet the level of therapeutic success could be improved significantly^30^. Clinical schedules for radical curative radiotherapy (with or without concurrent chemotherapeutics) deliver 66–70 Gy in 1.8–2 Gy fractions over 6–7 weeks^31,32^. Clinical regimens are delivered along such timescales to achieve greatest impact based on the five R’s of radiobiology: radiosensitivity, reoxygenation, redistribution, regeneration and repair^33^. Fractionation allows time for redistribution of cells through the cell cycle allowing treatment to occur during different phases to maximise effect, cells are most sensitive in G2 and mitosis^34^, fractionation further allows repair of sub-lethal injury in both normal and neoplastic cells. The ability to treat patient tumour on a chip and monitor the response thereof, provides scope to screen patients and predict radio-sensitivity/resistance; one of the 5 R’s not mitigated through the use of fractionation. Being able to select treatment based on radio-sensitivity would prevent patients with resistant tumours from undergoing unnecessary treatment with associated comorbidities, and reduce cost to health care providers (Fig. 1).

**Figure 2:**
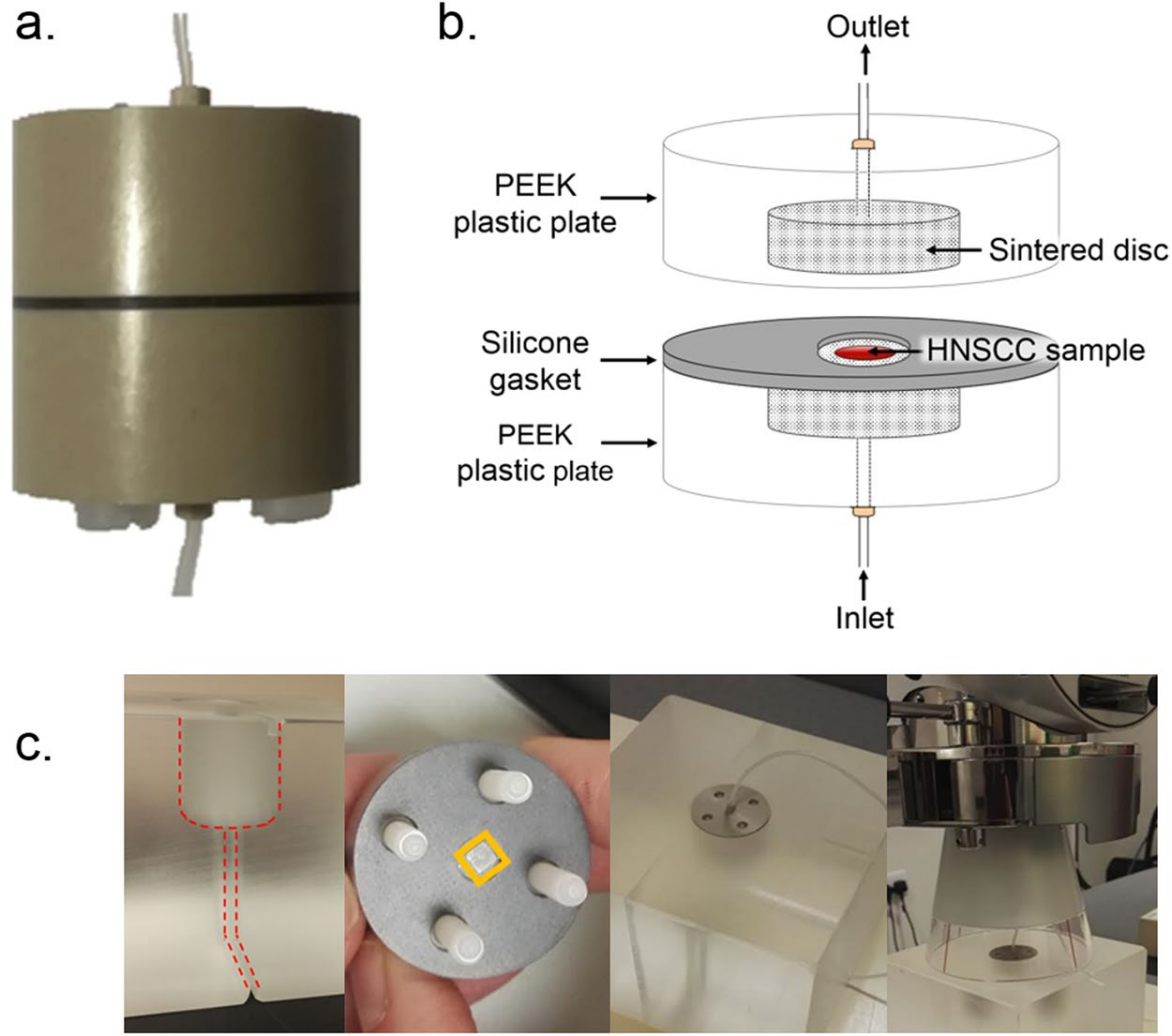
Tumour-on-a-chip Device. (**a**) Photograph of complete chip set-up. (**b**) Schematic of tumour-on-a-chip. All dimensions are shown in Supplementary Fig. S1. (**c**) Irradiation set up: PMMA phantom for tumour-on-a-chip device (milling outlined in red); placement of TLD or PCTS (outlined in yellow); Tumour-on-a-chip housed in phantom; XStrahl unit applied to phantom and device.

Herein, a unique tumour-on-a-chip device (Fig. 2 and Supplementary Fig. S1) is described that successfully maintains precision cut tumour slices (PCTS) in a viable state, mimicking *in vivo* microvascular flow^26,27^ to develop a highly reproducible and easy to use unit. A bespoke irradiation system accommodating the tumour-on-a-chip device has also been developed and characterised for highly-controlled irradiation, which can simulate the clinical dose regimen. To demonstrate that these tissue slices are affected by radiation and chemo-radiation treatments the effects on proliferation (Ki67 and Bromodeoxyuridine, BrdU), DNA damage response (phosphorylated H2AX) and caspase-dependent apoptosis (caspase-cleaved cytokeratin 18) were all successfully assessed. The robustness of the technology, and the ease with which standard methodologies can be integrated with the device offer a new way of informing the choice of therapeutic intervention.

## Results

### Rationale for tumour-on-a-chip system

The PEEK material selected for the device provided biocompatibility, mechanical strength and chemical stability. The device was designed to maintain sample integrity and improve ease of use over current systems^23,35^. A key design facet is “plug-and-play handling” essential for wide-spread adoption of the technology^36^. Issues of microchannel blocking have been mitigated through incorporation of sintered discs which encapsulate a network of micropores; blocking or leaking in the current system has never been observed. The sintered discs ensure even perfusion to all surfaces of the sample this is validated by fluid dynamic modelling (Supplementary Figs S2–S4 and Table S1).

### Off-chip analysis of tissue viability

**Figure 3:**
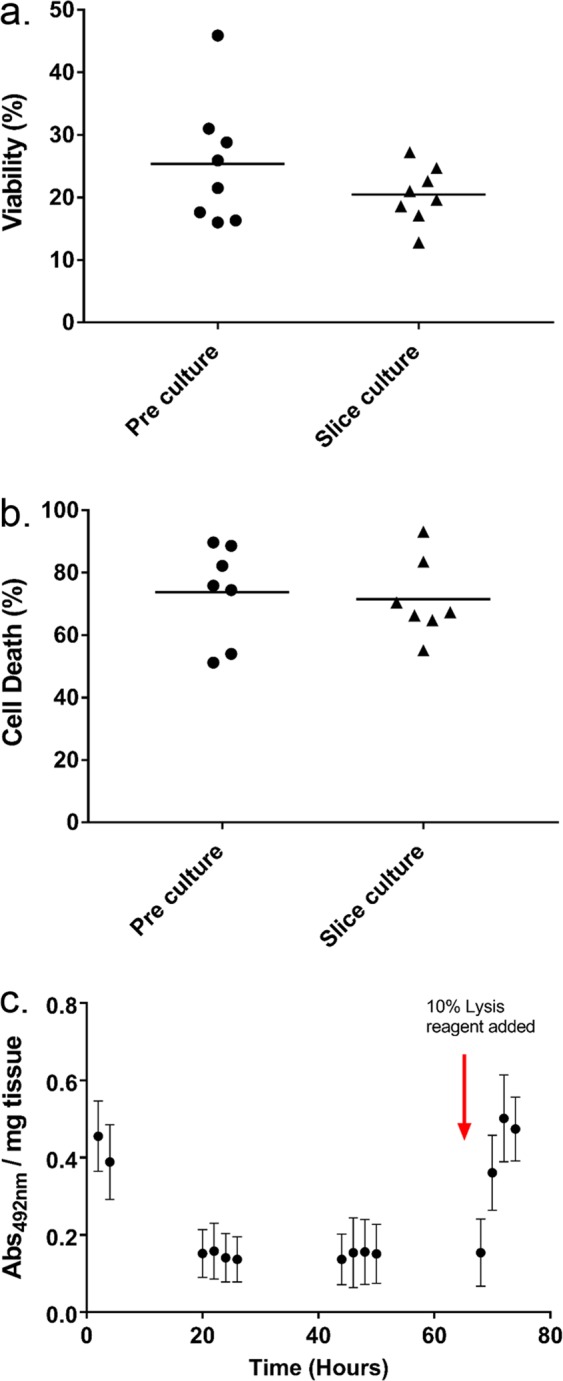
Viability of tumour samples prior to and following tumour-on-a-chip maintenance. (**a**) Viability of the tissue (%) from trypan blue exclusion (n = 8). (**b**) Cell death (Propidium iodide flow cytometry; n = 7). (**c**) Absorbance of formazan product (LDH release) measured in the effluent, red arrow indicates addition of 10% lysis reagent. Normalised per mg of tissue (n = 5).

**Figure 4:**
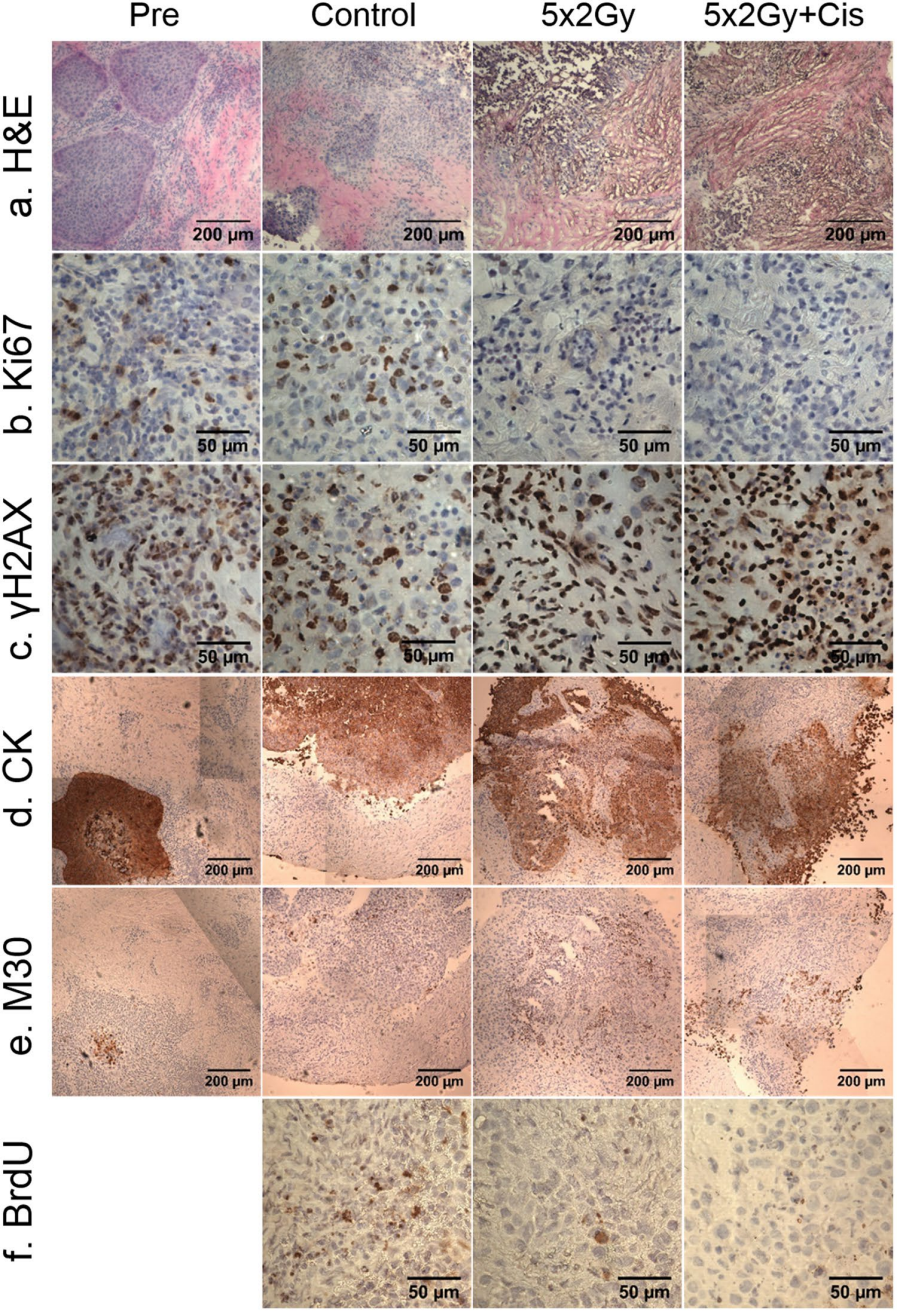
Laryngeal squamous cell carcinoma before and following on-chip culture with and without treatment. (**a**) H&E stained, x100 magnification. (**b**) Ki67 stained (proliferation), x400 magnification. (**c**) γH2AX (DNA damage response), x400 magnification. (**d**) Cytokeratin (CK: total tumour area), x100 magnification. (**e**) Caspase cleaved cytokeratin 18 (M30), x100 magnification. (**f**) BrdU stained (proliferation), x400 magnification. Representative images.

The viability of cells dissociated from the tissue (trypan blue exclusion) immediately following surgical resection (25.4% ± 3.6) was not significantly different to that of cells taken from tissue slices following 68 h culture (20.5% ± 1.6) on-chip (p = 0.112: paired t-test). Similarly, no significant difference in propidium iodide (PI) uptake (cell death), was observed (p = 0.767: paired t-test). Trypan blue and PI results were concordant, demonstrating the maintenance of cell viability within the HNSCC slices after perfusion on the tumour-on-a-chip system (Fig. 3a,b). Initial elevated LDH levels decreased to minimal levels 20 h into the culture period and remained low (Fig. 3c). Lysis reagent added at 68 h resulted in a sharp rise in LDH release. The morphology of tissue immediately following surgical resection and following 68 h culture showed maintenance of key tumour features including tumour islands and pale stromal areas with immune infiltrates (Fig. 4, H&E panel).

### Tissue morphology and IHC after on-chip treatment

Extensive dosimetry evaluation, to clinical specifications, confirmed a reproducible treatment environment for accurate delivery of 2 Gy fractions to HNSCC PCTS maintained in the novel tumour-on-a-chip/phantom set-up (Supplementary Figs S5–S7 and Tables S2–S4). A loss of tissue cohesion and degradation of nuclei (Fig. 4; H&E panel) was observed following treatment assault.

**Figure 5:**
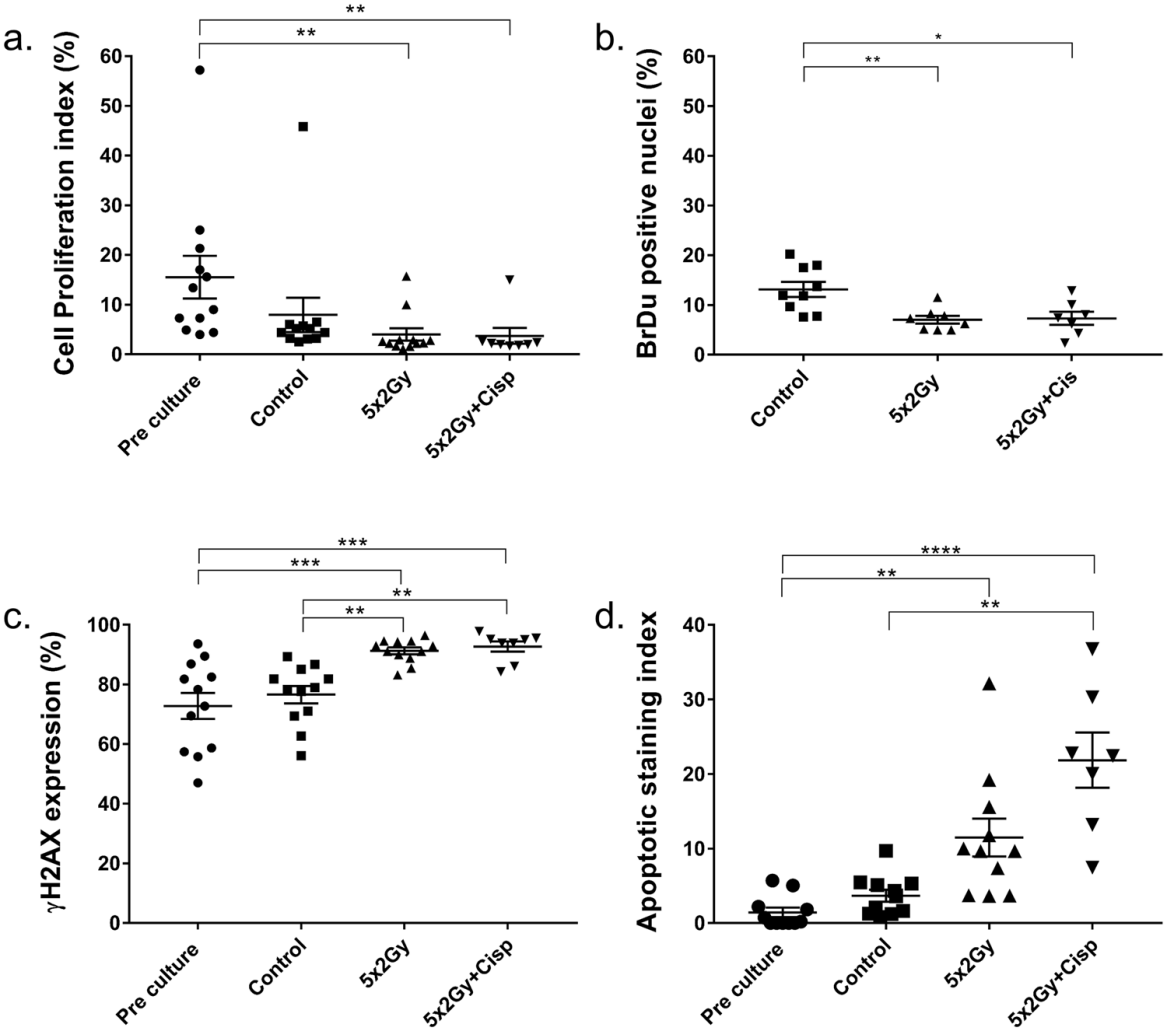
Immunohistochemical analysis of treated HNSCC PCTS. (**a**) Cell proliferation index (%) of the tissue, Ki67 positive nuclear area/total nuclear area x100. (Pre, control, 5 × 2 Gy n = 12, 5 × 2 Gy + cisplatin n = 8, Kruskal-Wallis). (**b**) BrdU positive nuclei (%; control n = 9, 5 × 2 Gy n = 8, 5 × 2 Gy + cisplatin n = 7, One-way ANOVA). (**c**) DNA damage response by positive γH2AX staining (Pre, control, 5 × 2 Gy n = 12, 5 × 2 Gy + cisplatin n = 8, One-way ANOVA). (**d**) Caspase cleaved cytokeratin 18 (Apoptotic index) by M30 antibody staining (Pre, control, 5 × 2 Gy n = 11, 5 × 2 Gy + cisplatin n = 7, Kruskal-Wallis). Mean + SEM shown. *p < 0.05 **p < 0.01 ***p < 0.001 ****p < 0.0001.

The Ki67 proliferation index was significantly lower following irradiation (4.0% ± 1.2) than in pre-culture tumour tissue (15.3% ± 4.3; p = 0.001; Figs 4b and 5a), however, the addition of Cisplatin had no effect. It is of note that the average Ki67 index decreased in the control sample (7.9% ± 3.5) relative to the pre-culture sample. Inter-tumoral heterogeneity of Ki67 index was observed, with pre-culture samples ranging from 4.0–57.2%; tumour sub-site and staging, together with the relatively small number of samples analysed are likely to be responsible for this (Supplementary Table S5). The incorporation of BrdU into replicating DNA^37^ (Fig. 4f) was found in 13.3 ± 4.6% of nuclei from control samples. Significantly lower BrdU staining was observed in treated samples (5 × 2 Gy = 7.0 ± 2.2%; p = 0.0064, 5 × 2 Gy + cisplatin = 7.3 ± 3.5%; p = 0.012). The levels of staining observed with Ki67 and BrdU are concordant (Fig. 5a,b).

Phosphorylation of H2AX at serine residue 139 (γH2AX) is one of the early events following the generation of DNA double strand breaks^38,39^ and was evident in all samples (Fig. 4c). The level of γH2AX expression was unexpectedly high in pre-culture samples (72.8% ± 15.1) but importantly, was not significantly different to control samples (76.6% ± 10.0). Significant increases in γH2AX expression were seen following both treatment regimens compared to pre-culture (5 × 2 Gy p = 0.003, 5 × 2 Gy + cis p = 0.005) and control (5 × 2 Gy p = 0.0043, 5 × 2 Gy p = 0.0052) samples (Fig. 5c).

No significant increase in apoptotic cells (cytokeratin/M30; Fig. 4d,e) within the tissue slices were observed following on-chip culture (3.7 ± 2.7%) compared with pre-culture samples (1.4 ± 2.1%; Fig. 5d). However, a significant increase in apoptotic cells was observed following 5 × 2 Gy with cisplatin (p = 0.0063) relative to control. Significant increases were also seen compared to pre-culture samples (5 × 2 Gy p = 0.0016, 5 × 2 Gy + cisplatin p < 0.0001). The addition of concurrent cisplatin, considerably increased the apoptotic cell fraction; with the concurrent treatment showing on average 1.9-fold higher apoptotic staining index relative to irradiation alone.

## Discussion

Maintenance of HNSCC PCTS within on-chip devices is in agreement with and extends previous studies^21,24,26,27,40,41^. In the first part of the work, tissue slices were dissociated to allow viability of cells immediately following surgical resection compared with those from tissue after 68 hours of culture on the on-chip platform. No significant difference was found in the percentage viability between pre- and post-culture tumour tissue, 25.4% (±3.6) and 20.5% (±1.6) respectively, as determined by trypan blue exclusion. Similarly, no significant difference in percentage cell death, as measured by PI uptake, was observed between the dissociated tumour cells before, 73.7% (±5.9) and after, 71.5% (±4.8) the 68-hour maintenance period. The concordance observed between viability (Trypan blue) and cell death (PI) results is in agreement with previous studies which utilised the same mechanical and enzymatic dissociation methods to assess the viability of HNSCC tumour samples before and following maintenance in a microfluidic culture device, for 48 hours, from a 5–10 mg piece of tissue^26^. A similar mechanical and enzymatic dissociation and flow cytometry approach was undertaken by Astolfi *et al*.^22^ to compare cell viability in micro-dissected tissues (300 µm thickness by 380 µm diameter) and “large tissue fragments” (8 mm^3^); they reported averages of approximately 45% and 22% respectively. The fact that significantly lower cell viabilities are observed when compared with assessments of the large tissue fragments is most likely due to the intrinsic nature of the assays, i.e. there is considerable handling involved in isolating the cells prior to cell staining and also since the entire sample is dissociated areas of dead cells (apoptotic or necrotic) may not be part of the slices obtained for immunohistochemistry but will be part of the cell sample that is analysed by flow cytometry; this is discussed at length by Astolfi *et al*. Furthermore, the reason the current study describes slightly lower viabilities (average 20.5%) is most likely attributable to the differences in tissue studied, i.e. in the current work they are patient-derived tumours and the previous work used xenografts from human cell lines. This study has highlighted the importance of studying both cell and tissue responses, appreciating the differences in results obtained from the distinct biological material.

Initial high LDH release from HNSCC samples on-chip are thought to be from damage to cells during slice preparation and the high release in response to lysis buffer indicates that cells with intact membranes are present following the 68 h maintenance period; this is in agreement with previous findings^21,27,42^. Maintenance of morphology correlates with others who demonstrated *ex vivo* maintenance of PCTS (primary patient, cell and patient derived xenografts, and cultivated on a filter support) by H&E staining^43^. Ki67 staining has validated a microfluidic chip for maintenance of human intestinal tissue (72 h)^21^; images correlate with staining levels displayed here and indicate a low basal proliferation (estimated <20%). It is postulated that the samples which saw a proliferation decrease following on-chip maintenance are inherently more sensitive to changing conditions, and therefore may be more sensitive to treatment. BrdU has been used previously as a proliferation marker in on-chip platforms demonstrating proliferation of mouse embryoid bodies^35^ and successfully demonstrated a reduction in incorporation following treatment in the current study.

Degradation of nuclear morphology following chemotherapeutics has been previously demonstrated^44^ mirroring our treated samples. The analysis of multiple markers within this study demonstrated tissue response to therapeutic assault. γH2AX levels increase immediately following radiation exposure, returning to basal levels within 6–24 hours^45,46^; hence samples were snap frozen 1 hour after the final irradiation fraction and as such, induction of DNA damage response following radiation was observed. The presence of a basal level of γH2AX staining in baseline and untreated tissue on the microfluidic device was in agreement with previous studies which have found more γH2AX positivity in untreated archival endometrial tumour tissue compared with non-cancerous endometrial tissue suggesting the DDR pathway is already activated to an extent in cancerous tissues^47^. Response to irradiation has historically been measured in terms of measuring the activation of the DNA damage response pathway by counting an increase in the number of γH2AX positive foci in the nuclei of cells. However, the vast majority of these studies have been performed using cell lines or dispersed primary tissue. Although the γH2AX foci were clearly evident in cells within the HNSCC tissue sections in this study the tissue contains a plethora of different cell types and as a result there was variation in the clarity of the foci due to cellular heterogeneity, thus cells with positive nuclei containing more than one foci were enumerated as done previously^48^. Furthermore, diffuse staining in tissue has been reported previously Akbay *et al*. and Banuelos *et al*. who clearly showed foci in individual cells from cell lines and dispersed xenograft tissue, but the foci were less defined when whole sections of cervical tissue were immunostained^47,48^.

The use of γH2AX following *ex vivo* irradiation has previously been shown on primary tumours from multiple origins; with residual foci (24 hours post treatment) in line with expected differences in radiosensitivity from different tumour types^49^. It was hypothesised that since, cisplatin treatment causes collapse of the replication fork and subsequent double strand breaks, a further increase in γH2AX would be observed but this was not the case. Previously the assessment of residual γH2AX foci in cisplatin treated xenograft tumours was analysed 3 days following treatment^48^; it is possible no effect was observed here with the addition of cisplatin because of the short analysis time frame. In contrast, a significant increase in caspase-dependant apoptosis was demonstrated with the addition of cisplatin; extending previous work which showed a significant increase in the amount of apoptosis occurring in a fractionated (5 × 2 Gy) treatment compared to a 2 Gy single dose in HNSCC samples^24^. Cisplatin undergoes conversion in the cytoplasm of a cell to its aquated derivative (*cis-*[PtCl(NH_3_)_2_(OH_2_)]^+^) which reacts with DNA to form a bi-functional adduct, inhibiting replication and initiating apoptosis^50^.The presence of caspase-cleaved cytokeratin 18 as a marker of apoptosis initiation has also been shown following chemotherapeutic treatment in gastro-oesophageal cancer^51^ and rectal cancer^52^.

Inter-tumoral heterogeneity was evident in all markers investigated, and has also been observed in evaluating the effect of targeted agents on HNSCC PCTS using Ki67 previously. The authors noted variation in the basal proliferative index between 25–45%^53^. Inter-patient variation in Ki67 was further shown following single fraction irradiation by Cheah *et al*., in 5 primary HNSCC samples^27^. Additionally, Chatzkel *et al*., reported a baseline Ki67 expression of 16–97% in 59 archived HNSCC which did not correlate with patient response to induction chemotherapeutic regimens^54^.

The ability to investigate tumour biology and response to therapeutic assaults in the context of tumour tissue itself is a facet lacking in many cell based *in vitro* models^43^. Others have demonstrated on-chip *ex vivo* tissue systems for chemo-sensitivity testing^22,55^. However, previous work has been largely based on xenograft tissue with only Astolfi and colleagues progressing to the use of 4 patient samples, from 3 different malignancies, to validate viability, with a single ovarian cancer sample used in chemo-sensitivity showing a reduction in viability following carboplatin treatment (assessed by confocal microscopy). Irradiation of head and neck tumour on-chip has been shown previously, with associated increases in apoptotic cell fraction following treatment^24,27^.

The PEEK device used in the current study has significantly improved on the previous extensively used glass design^24,27,40^; the new device has proven to be 100% reliable in set-up and operation, is easy to use, and has improved flow kinetics resulting in uniform delivery of nutrients to the PCTS. Further, a bespoke irradiation system allowed for precise and reproducible treatment delivery at depth; this coupled with the analysis of multiple markers demonstrated monitoring of patient tissue response to irradiation *ex vivo*. To our knowledge this is the first on-chip platform to demonstrate chemo and radio-therapeutic assault concurrently; critically it has allowed human primary samples to be assessed in this manner which gives clinical utility to the technology.

To further advance the clinical utility of the bespoke on-chip system and irradiation jig presented here, a multi-centre clinical trial is now required. This would allow evaluation of patient biopsies on-chip with appropriately scaled treatment modalities mimicking the exact clinical regimens being administered to the patients at the same time as alternative options, i.e. potential second or third line chemotherapy. Critically, this trial would have prolonged patient follow up, that would allow correlation between clinical outcome and on-chip data generated within 3–7 days. The study would need to be large, involving multiple chips of each treatment / patient, and appropriately powered to ensure intra- and inter-patient heterogeneity was appropriately considered. The trial would provide validation of the utility of data from the tumour-on-a-chip platform for informing patient treatment prior to commencement, when it is most likely to be effective. The current study has focussed on HNSCC however the platform is applicable to other solid malignancies. The ease of use and robustness of the device represent a significant advancement for *ex vivo* tissue studies, with widespread applications in research, clinical and drug development settings.

## Materials and Methods

### Design and fabrication of an on-chip culture system

Each tumour-on-a-chip platform is composed of two identical PEEK parts (Polyetheretherketone, 30 mm diameter, Direct Plastics, Sheffield, UK; Fig. 2) with central axial threaded holes to secure the ETFE tubing (Ethylene-tetrafluoroethylene, 0.8 mm internal diameter; IDEX Health & Science, Cambridge, UK) to the system via coned adaptors (LabSmith, Mengel Engineering, Denmark). A cylindrical recess accommodates porous sintered Pyrex discs (Pore size index 160–250 µm, Supplementary Table S1; The Lab Warehouse, Grays, UK). The central gasket (1 mm Silicone) overlaps the sintered disc by 2 mm generating a central tissue well (6 mm diameter) ensuring a no-flow pathway around the disc and generating a flat front of fluid flow approaching the tissue sample. The PEEK plates and gasket contain 4 screw holes for assembly (Fig. 2, Supplementary Fig. S1).

### Patient biopsies and slice preparation

Tumour samples (n = 18; Supplementary Table S5) were taken from HNSCC patients after obtaining written informed consent; prior to commencement, approval for the work was obtained from the Yorkshire and Humber Local Research Ethics Committee (LREC-10/H1304/6) and NHS R&D (R0987) department. All research was performed in accordance with relevant guidelines and regulations.

Recruited patients had no previous cancer diagnosis. Samples were processed within 90 minutes of excision by vibratome slicing in ice-cold PBS (350 μm slices, 0.1 mms^−1^ and amplitude 2 mm; Leica VT1200S, Milton Keynes, UK). Slices were punched to 5 mm diameter (Biopsy punch; Stiefel, Middlesex, UK) to generate a precision cut tumour slice (PCTS) that was weighed before loading into the device.

### Device set up

Dulbecco’s Modified Eagle’s Medium (DMEM, Lonza, Slough, UK) was supplemented as previously described^26^, loaded into a 10 ml syringe and connected to the 2-part adapter and ETFE tubing via a 0.22 µM filter (Millipore, Watford, UK). PCTS were loaded into the tissue well on top of a 70 µm nylon membrane (BD, Oxford, UK) before assembly of the device. The system was connected to a Harvard PhD2000 syringe pump (Harvard, Cambridge, UK), and was maintained at 37 °C with pressure driven flow at a volumetric flow rate of 2 µlmin^−1^. Each device was perfused for 68 h with effluent collected at 2 hourly intervals. Bromodeoxyuridine (BrdU; 10 µM) was added for the final 16 h of perfusion.

### Off-chip analysis of dissociated cells

Dissociation of tumour tissue prior to and following on-chip culture (n = 11, from 8 tumours) was achieved by a combination of mechanical mincing of the tissue and enzymatic incubation (0.024% w/v Collagenase IV; Sigma-Aldrich: Dorset, UK and 0.02% w/v DNase I; Roche, Herefordshire, UK) in complete medium for 2 hours under constant rotation at 37 °C and 5% CO_2_. The resultant cell suspension was passed through a 70 micron cell strainer (BD Biosciences) and centrifuged to pellet the cells (400 × *g* for 5 minutes). The pellet was resuspended in 1 ml of complete medium before the percentage viability of the tissue was determined by trypan blue exclusion. Flow cytometry was also used as an end point measurement of cell death (n = 9, from 7 tumours) which was quantified by propidium iodide (PI; Sigma-Aldrich) staining. Briefly, cells were rinsed in PBS (Oxoid, Thermo Scientific, Hampshire UK)/BSA (2.5 g/l; Thermo Scientific)/Azide (0.0624% (v/v) Sigma-Aldrich) before staining in 100 µl total volume with 500 µg ml^−1^ PI. Following a further wash, cells were acquired on a FACSCalibur flow cytometer (BD Biosciences) and data were analysed using Cell Quest Pro Software, version 6.0.

### Measurement of lactate dehydrogenase release

The effluent (n = 8, from 5 tumours) was collected at 2 hr intervals and stored at 4 °C until the end of the culture time period, at which point 10% lysis reagent (Roche, UK) in complete medium was perfused through the system at 2 µlmin^−1^ for a further 6 h. The LDH assay (Cytotoxicity Detection Kit Plus, Roche, UK) was conducted following the manufacturer’s protocol and results expressed as an average of duplicate readings normalised per mg of starting tissue.

### Tissue embedding and morphological analyses

Tumour samples were embedded both prior to and following on-chip culture in Tissue-Tek OCT (optimum cutting temperature; Sakura, Berkshire, UK) embedding medium by freezing in liquid nitrogen-cooled 2-methylbutane (Sigma-Aldrich). Tissue sections (8 µm) were cut and fixed for 20 minutes in −20 °C cooled methanol before standard Haematoxylin and Eosin staining^26^.

### Irradiation of tumour slices ± concurrent Cisplatin

Irradiation (n = 12) was performed using a 120 kVp photon beam (Half-Value Layer = 5.0 mm Al) produced by an XStrahl 200 superficial unit. The devices were irradiated within a custom-made Perspex (PMMA) phantom (dimensions: 12 × 12 × 8 cm^3^) providing a reproducible setup and a full-scatter condition (for kV energies) simulating clinical cases where cancerous tissue is irradiated at depth (Fig. 2c and Supplementary Fig. S5). Samples received 10 Gy in 5 × 2 Gy fractions in a three-day schema ensuring a gap of >6 hours between fractions on the days of twice-daily exposures (dosage and gap typical of clinical regimens).

The machine output setting, in the form of monitor units (MU), for the specific beam quality/setup, that would result in tissue samples receiving the prescribed dose per fraction was established using lithium fluoride thermo-luminescence dosimeters (TLD) in a four-step calibration process using a Harshaw TLD model 5500 automatic reader. An overall dosimetric accuracy of ±5–10% was pursued (Supplementary Figs S5–S7 and Tables S2–S4).

Cisplatin (2.5µgml^−1^ based on blood plasma concentrations^56^; Acros Organics, Loughborough, UK) was added to complete medium for the final 48 h of culture alongside the 5 × 2 Gy irradiation fractions.

### Immunohistochemistry for radiation response markers

Frozen tissue sections (8 µm) were fixed in −20 °C methanol and subjected to immunohistochemistry staining^24,27^. Ki67, BrdU, γH2AX, M30 and cytokeratin antibodies were used (Supplementary Table S6). Imaged slides were quantified for Ki67, BrdU and γH2AX using 5 randomly selected fields of view (x400 magnification). The Ki67 and BrdU nuclear staining indices (positive/total nuclei x100) were quantified using Immunoratio (version 1.0c)^57^ for ImageJ^58^. γH2AX stained nuclei (those with one or more foci) and total nuclei were manually counted on ImageJ and percentage positivity determined. A tiled whole tumour section image was constructed (x100 magnification) for quantification of M30 and CK staining on serial sections. The staining index of cleaved cytokeratin 18 was determined as the apoptotic area (total area M30 positive staining/total area CK positive staining) × 100.

### Statistical analyses

All values are reported as mean + SEM unless otherwise stated. Statistics were performed in GraphPad Prism 6 (GraphPad Software, Inc., La Jolla, CA USA). All data sets were tested for normal distribution using the D’Agostino & Pearson normality test. Multiple comparisons were analysed by one-way ANOVA with post-hoc Tukey tests. In the event that the data was found not to be normally distributed the Kruskal-Wallis test of average with Dunn’s multiple comparisons tests was used. Paired t-tests were used to analyse data containing only two groups. Data were considered significant at the 95% confidence interval (p < 0.05).

## Supplementary information

Supplementary Figures and Tables

Supplementary Fig S4 Video
