# Supplementary material for: A patient tumour-on-a-chip system for personalised investigation of radiotherapy based treatment regimens
Source: Sci Rep. 2019 Apr 19;9:6327. doi: 10.1038/s41598-019-42745-2 (PMC6474873; doi:10.1038/s41598-019-42745-2)
Supplement: Supplementary file 1 — Supplementary Figures and Tables [file 41598_2019_42745_MOESM1_ESM.docx]

**Supplementary Information**

**A patient tumour-on-a-chip system for personalised investigation of radiotherapy based treatment regimens**

R. Kennedy PhD,^a^ D. Kuvshinov PhD,^b^ A. Sdrolia MSc^c^, E. Kuvshinova PhD^d^, K. Hilton MPhys MSc^c^, S. Crank FRCS^e^, A. W. Beavis PhD^acf^, V. Green PhD ^a^, and J. Greenman PhD^a*^

**
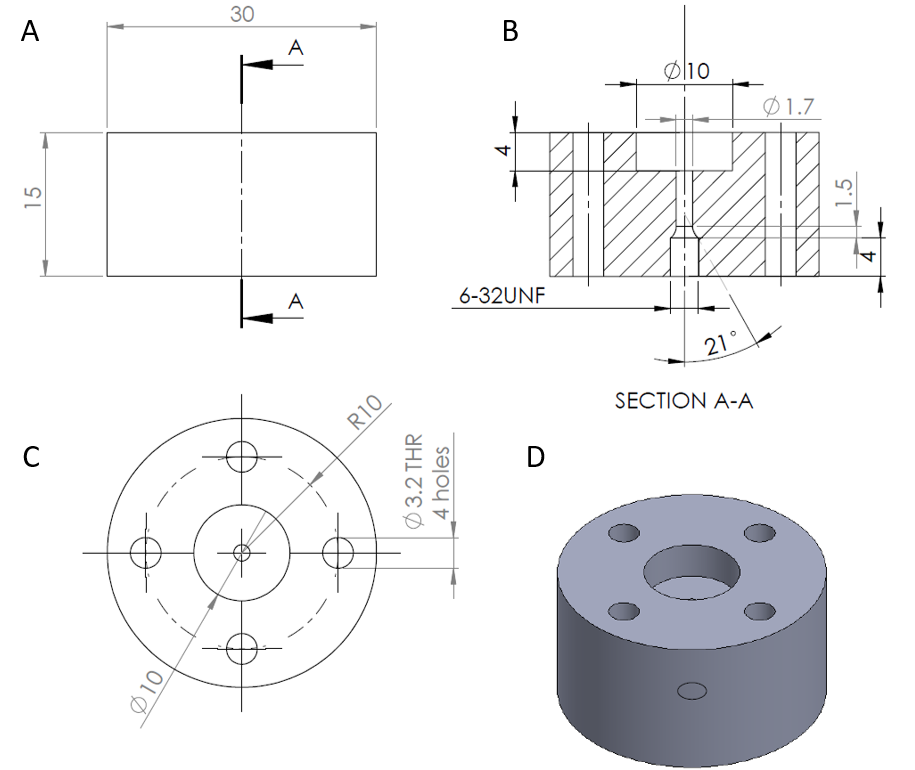
**

**Supplementary Figure S1:** Computer assisted design (SOLIDWORKS, Waltham, MA, US) drawings of PEEK plate for tumour-on-a-chip device. (a) Side view showing cutline for (b) Internal geometries of inlet, recess for sintered disc, axial threaded holes for interface with tubing adaptor. (c) Top view showing diameter of recess and threaded screw holes. (d) Filled drawing of a single PEEK plate. Dimensions in mm. Ø, diameter.

**
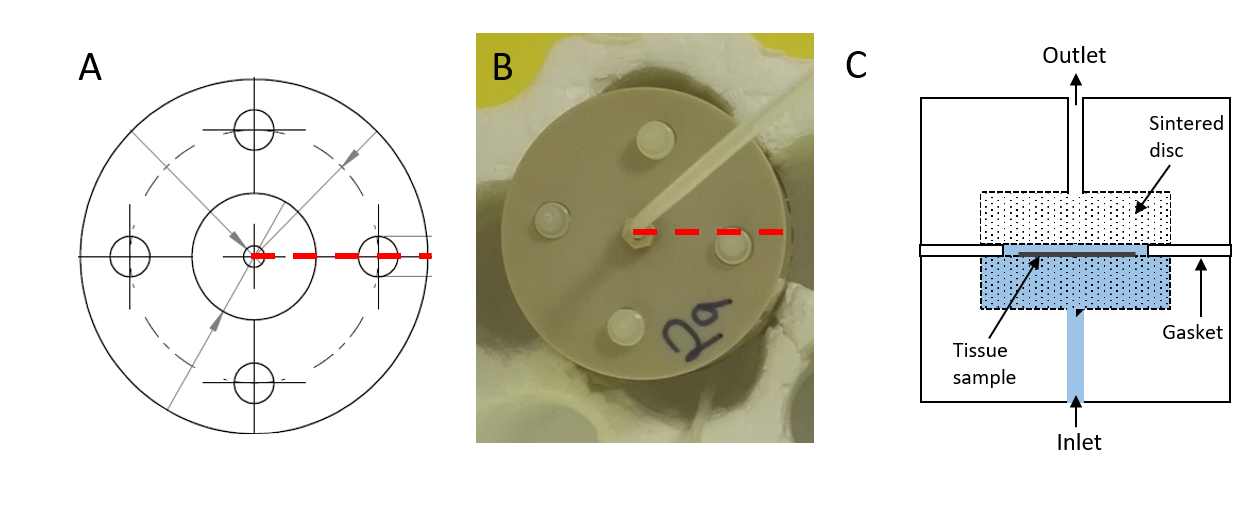
**

**Supplementary Figure S2:** The cut line of plane modelled by computational fluid dynamics of tumour-on-a-chip device (a&b). (c) internal components of device: blue shading highlights the area modelled for fluid dynamics (sample culture area).


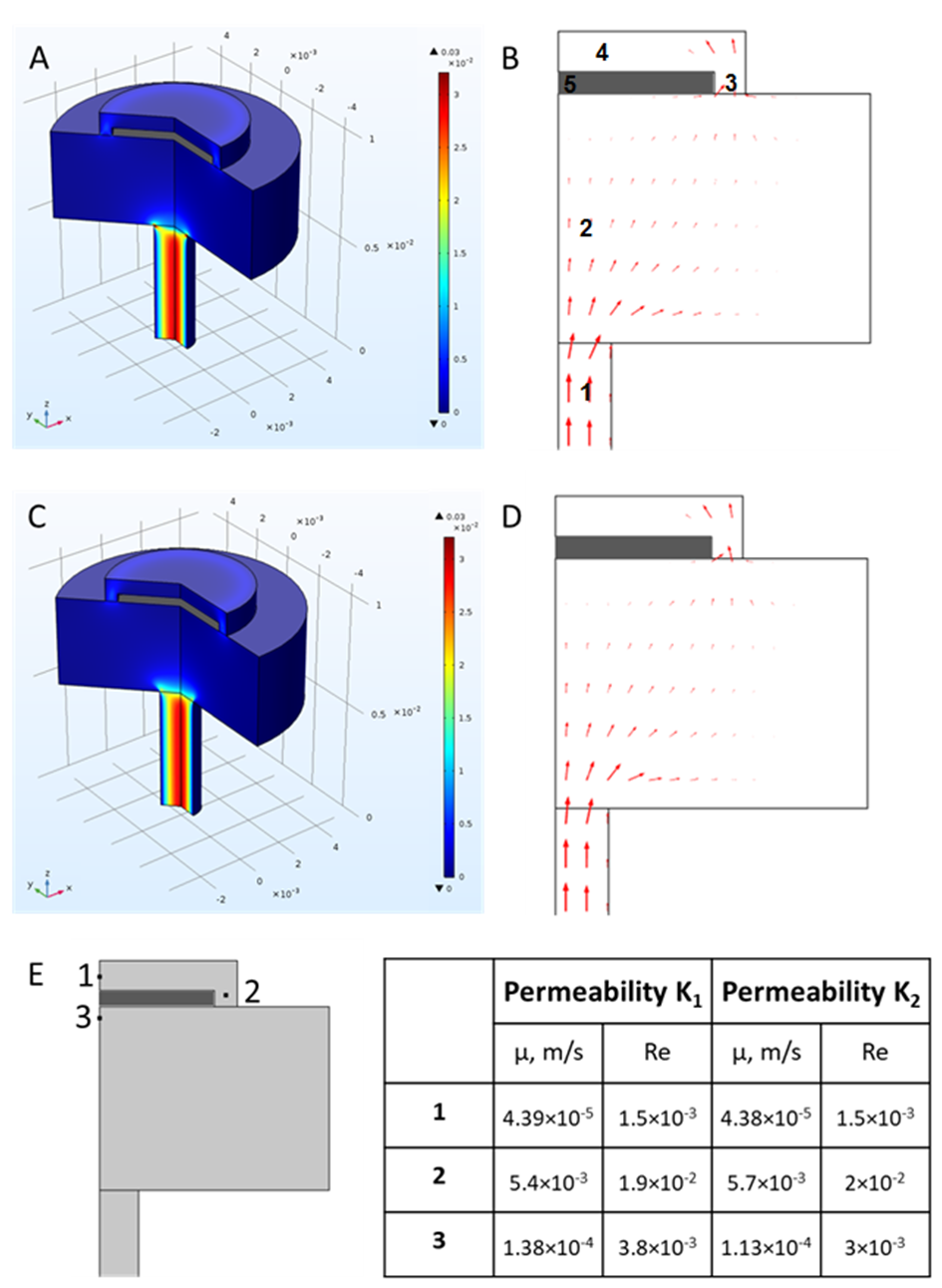


**Supplementary Figure S3. Computational Fluid Dynamics of Tumour-on-a-chip device.** Computer simulation of fluid flow in the perfusion chamber and around the sample was performed using COMSOL Multiphysics software, version 5.2a (COMSOL, Cambridge, UK). A built-in "Free and porous medium flow" module allowed capillary flow through a membrane to be coupled with pressure driven flow. **(**a) The velocity field surrounding the central tissue well for K_1_ permeability (1x10^-11^ m^2^), streamlines are also shown. (b) The cross section consists of bottom inlet (1), the volume of the bottom sintered disk (2), the side gap between the sample and the gasket (3), and the top gap between the sample and the top sintered disk (4). The biological sample (5) is set as solid non-permeable component of the flow path. **(**c) The velocity field surrounding the central tissue well for K_2_ permeability (1x10^-9^ m^2^), streamlines are also shown (d). (e) The points studied for calculation of Reynolds number and velocity of fluid (v) are indicated numerically (1-3), values for both permeabilities are tabulated. Placement of PCTS is indicated by dark grey shading, the area modelled for fluid flow is indicated by light grey shading. Component parts of tumour-on-a-chip device are labelled as in figure S2C.

The liquid flow path selected for modelling (Fig. S3) consists of bottom inlet (1), the volume of the bottom sintered disk (2), the side gap between the sample and the gasket (3), and the top gap between the sample and the top sintered disk (4). The biological sample (5) is set as a solid, non-permeable, component of the flow path in the models as any flow through the tissue is negligible in relation to the flow stream. The bottom of the top sintered disk was set as the outlet for the liquid. The cross section of the flow pass is presented in Fig. S3b. The selection permitted numerical investigation of the flow patterns in the area of interest around the biological sample.

The glass sintered discs used in the chip presented an array of cylindrical pores of irregular diameters and length, due to the manufacturing method. The sintered disc used in the given device was used to dissipate fluid flow resulting in homogeneous conditions throughout the tissue chamber. Development of the bulk structure of the sintered disc in the model is limited by the computer capacity, thus a porous matrix with defined properties was realised in the model. The Navier-Stokes equation (1) describes a free flow, and Brinkman equation (2) describes a flow in the porous medium. Both formulae are engaged to solve continuous pressure and velocity fields along the tissue chamber.

$\rho\left( \boldsymbol{u}\cdot\nabla\right)\boldsymbol{u}=\nabla\cdot[-p\boldsymbol{I}+\mu\left( \nabla\boldsymbol{u}+\left( \nabla\boldsymbol{u} \right)^{T} \right]+F$ (Equation 1),

where ρ - density, $\boldsymbol{u}$ - velocity vector, $p$ - pressure, I - identity matrix, μ - dynamic viscosity, T - transparent matrix, F - body force, $\nabla$- del operator.

$\rho\nabla\boldsymbol{u}=\nabla\cdot[-p\boldsymbol{I}+\mu\left( \nabla\boldsymbol{u}+\left( \nabla\boldsymbol{u} \right)^{T} \right]-\frac{\mu}{K}\boldsymbol{u}+F$ (Equation 2),

where K - medium permeability.

Conservation of mass equation:

$\rho\nabla\cdot\boldsymbol{u}=0$ (Equation 3),

K=f(ɛp) (Equation 4),

f is a function, describing the dependence of permeability on the porosity of the sintered disc (ɛp).

In order to characterize the flow type (laminar or turbulent), the Reynolds number, Re (5) at different flow points was calculated using parameters of the experiment.

$Re=\frac{\rho\boldsymbol{u}L}{\mu}$ (Equation 5),

where L - characteristic linear dimension.

The calculation of governing partial differential equations (PDE) was implemented by using the finite element method, and meshed by physically controlled fine mesh, constituting mainly quadrilateral and triangle form to a total of 6250 elements. Input rheological parameters for simulation were established as follows: T=37°C, viscosity of culture medium was μ=6.9×10^-4^ Pa·s, inlet boundary condition was set as volumetric flow rate of 2 μl/min converged taking into account the inlet width of 1.7 mm, outlet boundary condition was set as open to atmosphere, P=0. The liquid in the reactor was defined as Newtonian liquid, having constant dynamic viscosity along all exhibited shear rates. Porosity as a ratio of void part of the sintered disc to the bulk volume of the sintered disc was calculated in the laboratory and was equal to 0.24 (Table S1). The second parameter, which characterizes properties of the sintered disc is permeability, this demonstrates the capability of a porous medium to pass gases or liquids. Permeability depends on configuration of void fractions in the sintered disc. Due to the method of manufacturing the sintered disc (glass powder sintering) it is not possible to control formation of a void fraction, so for the purpose of computer simulation the permeability was taken equal to: K1=1×10^-11^ m^2^ and K2=1×10^-9^ m^2^, based on measurements made for sintered discs with different pore configuration 1. The PCTS was modelled as a no-slip boundary:

K=0, $\boldsymbol{n}\cdot\boldsymbol{u}=0$, (where n = normal vector to the surface). The area resolved for fluid flow is shown graphically (Fig. S2c).

The velocity field within the tumour-on-a-chip device was modelled for permeabilities K_1_=1×10^-11^ m^2^ (Fig. S3a) and K_2_=1×10^-9^ m^2^ (Fig. S3c). Streamlines for permeabilities K_1_ (Fig. S3b) and K_2_ (Fig. S3d) are also displayed. It can be seen that for K_1_ and K_2_, permeabilities that flow within the system are laminar with the velocity value uniform throughout the volume of the glass sintered disc.

Numerical values of Reynolds number and velocity are provided for 3 points 0.25 mm away from the tumour slice boundary (Fig. S3e). The Reynolds numbers obtained are values characteristic of laminar flow at the K_1_ and K_2_ permeability conditions. Several conclusions can be drawn following the computer simulation of the fluid flow within the tumour-on-a-chip platform comprising a porous medium for liquid supply. Firstly, permeability of the sintered disc does not affect flow velocity field, with minimal changes observed in velocity values. Further, due to the axisymmetric configuration, the chip provides a uniform and controlled flow. Moreover, the fact that the flow in the chip is directed along the axis of the sample, excludes the possibility of tissue deformation.

**Supplementary Figure S4. Video simulation of flow in the device**

The design of the setup is made to insure constant uniform flat front flow of the media as it approaches the surface of a sample. The flow pattern became fully established within 0.22s from the injecting moment. There is no flow velocity fluctuation in the region of the sample. The pattern of the obtained stream lines confirms that the presence of the porous structure resulted in a practically flat front of media approaching the sample surface with the expected stagnation point with the radii of 0.12 mm in the centre of the round shaped sample.

**
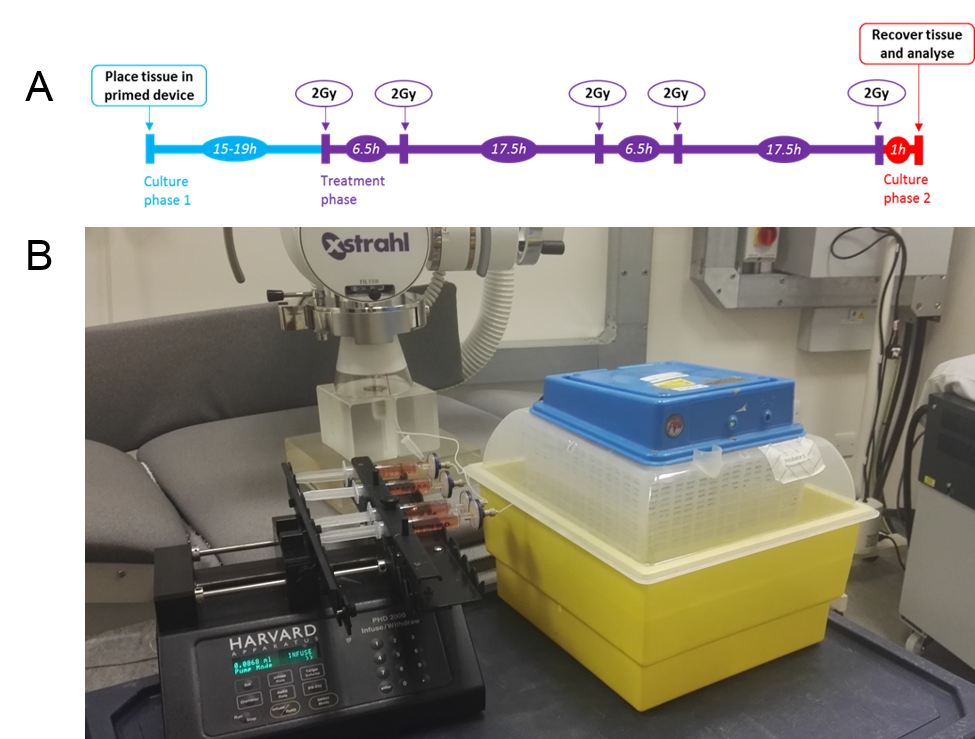
**

**Supplementary Figure S5:** Irradiation system set up. (a) Tissue irradiation timeline, in cases treated with concurrent cisplatin, drug is added for treatment phase and culture phase 2 (purple and red lines). (b) Image of pressure driven syringe pump (Harvard Apparatus) and incubator housing parallel tumour-on-a-chip devices, phantom and XStrahl superficial irradiator allowing irradiation treatment whilst under continuous perfusion.

Prior to TLD calibration, an output measurement was performed on the XStrahl unit using a Farmer chamber (in air, as per the local QA protocol) to ensure that it is within clinical tolerance (± 2%^2^). A brown solid water sheet with TLD cut-out was used (10mm, Fig. S6a), placed on top of sufficient backscatter material (10 cm of plastic water). Five, previously annealed, TLDs were placed in the recesses closest to the beam’s central axis and then exposed to 2 Gy (200 MU) delivered at 0 cm depth using a 5 cm diameter open applicator 20 cm FSD (local reference conditions). This process was repeated to take into account any small positional variations therefore the mean reading of the two exposures was considered per TLD. The TLDs were read in a Harshaw 5500 TLD reader using a high gain time temperature profile (TTP) protocol (Fig. S6).


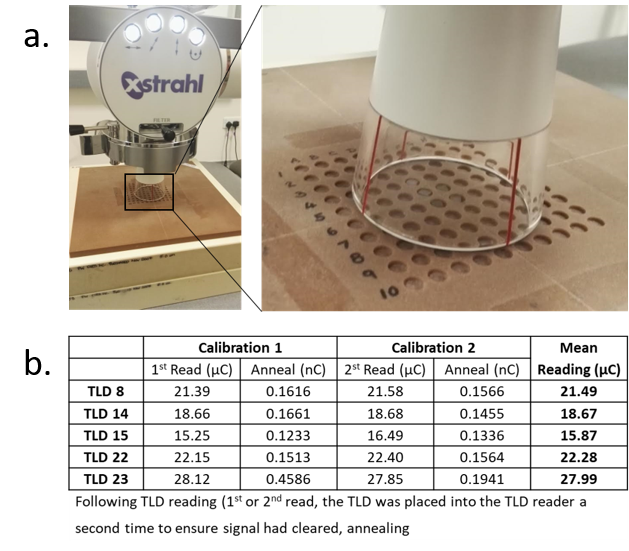


**Supplementary Figure S6: (**a) At surface TLD calibration in solid water sheet (reference conditions). (b) TLD calibration values


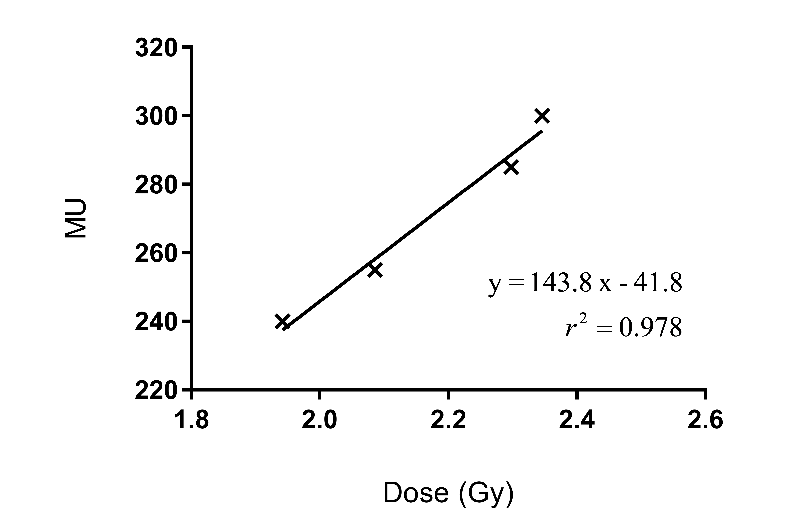


**Supplementary Figure S7:** Absorbed dose vs. MU delivered for TLDs 8, 14, 22 and 23.

**Supplementary Table S1:** Calculation of glass sintered disc porosity

| **Sintered disc** | **Dry mass**  (g) | **Saturated mass** (g) | **H_2_O mass**  (g) | **H_2_O volume / Disc volume** |
| --- | --- | --- | --- | --- |
| 1 | 0.3993 | 0.4750 | 0.0757 | 0.241 |
| 2 | 0.3976 | 0.4728 | 0.0752 | 0.239 |
| 3 | 0.3942 | 0.4663 | 0.0721 | 0.230 |
| **Calculated porosity of sintered disc** | | | | **0.237** |
| Glass sintered discs (n=3) were weighed (dry) on a high precision balance, then immersed in ultra pure water before being sonicated to remove any residual air. Discs were re-weighed in order to calculate the water mass (hence volume) of the void fraction. The volume of the glass sintered disc is 0.314 cm^3^. | | | | |

**Supplementary Table S2:** TLD absorbed dose per delivered MU


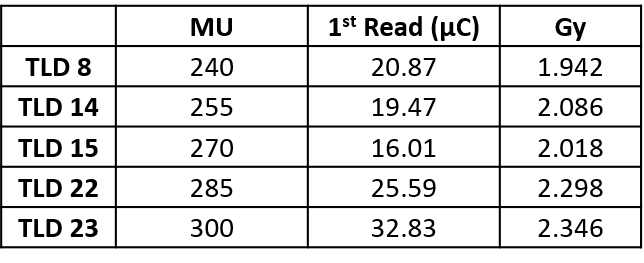


Each TLD was placed in the same tumour-on-chip device and exposed to 240-300 MU; the reading in µC was divided by the mean calibration reading of the same TLD (Gueven *et al.* 2017), then multiplied by 2 to account for the calibration dose of 2Gy. For example, for TLD 8: (20.87/21.49) x 2 = 1.942 Gy (Mayles et al. 1999). TLD 15 exhibited an under-reading acting as an outlier; this could be attributed to a random error such as experimental setup and reading errors; thus, it was excluded from the graph (Fig. S7). The results were plotted with the data from TLD 15 omitted (R² = 0.978; Allisy-Roberts and Williams 2008). In this case, the interpolation estimated number of MU needed to deliver an absorbed dose of 2 Gy at the desired depth is: 143.8 x 2 – 41.8 = 245.8 MU using the derived equation of the line.

**Supplementary Table S3:** TLD readings following 246 MU exposure in the same tumour-on-a-chip device.


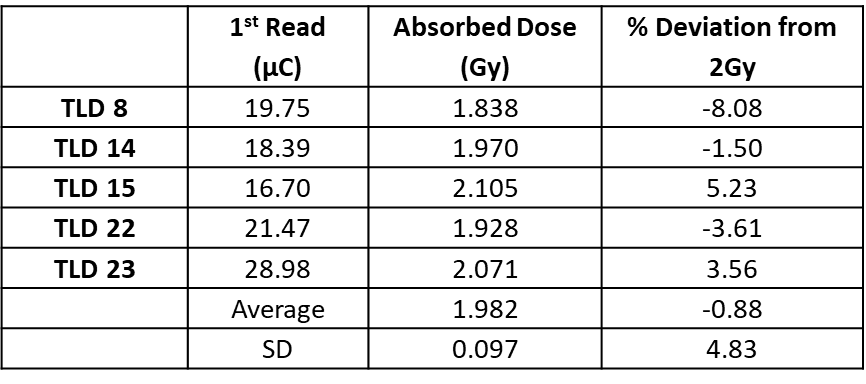


Following annealing, 246 MU were delivered independently to each of the 5 TLD in the same chip device. Given that the average absorbed dose received by all the 5 TLD was 1.982 Gy, the delivered MU was scaled by a factor of 2/1.982 (equivalent to 248 MU) in order to yield a mean absorbed dose of 2 Gy.

**Supplementary Table S4**: Dose received by TLD 8 in three different tumour-on-a-chip devices.


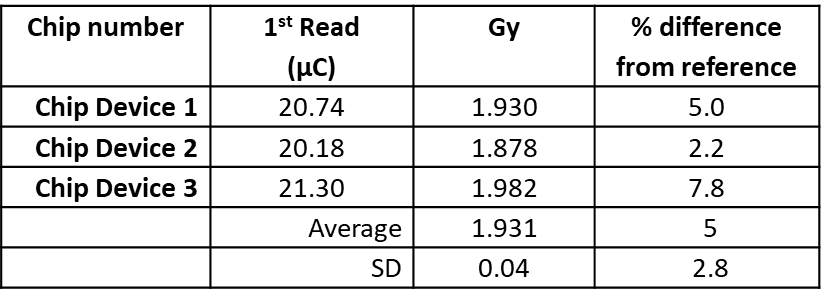


In order to check variation of dose delivered between devices the same TLD (TLD 8) was placed in 3 separate devices and was exposed to the same number of MU (246). The average deviation from the reference measurement, also taken at 246 MU (19.75 µC, Table S4 ; TLD 8) for the three sample devices was 5% which is within acceptable levels of uncertainty, particularly given the inherent uncertainty of TLD 3.

**Supplementary Table S5:** Clinical information for 18 patient samples utilised within this study

| **Primary site** | **Tumour stage** | **Patient age** | **Gender** | **Study element** | **Number of devices for viability study** | | **Number of devices for IHC** | | | |
| --- | --- | --- | --- | --- | --- | --- | --- | --- | --- | --- |
|  |  |  |  |  | **Pre-culture** | **Slice culture control** | **Pre- culture** | **Slice culture control** | **5x2Gy** | **5x2Gy+Cis** |
| Left Tonsil | T2N0 | 56 | M | V | 1 | 2 | - | - | - | - |
| Larynx | T4aN2b | 73 | M | V | 2* | 2* | - | - | - | - |
| Larynx | T4N0 | 61 | M | V | 1 | 1 | - | - | - | - |
| Right Mandible | T1N2b | 55 | M | V | 1 | 1 | - | - | - | - |
| Parotid | unknown | unknown | unknown | V | 2 | 2 | - | - | - | - |
| Tongue | T1/2N0 | n/a | M | V | 1 | 1 | - | - | - | - |
| Tongue | T3N1 | 69 | M | V, R, C | 3 | 1 | 1 | 2 | 1 | 1 |
| Left retromolar and mandible | T4N0 | 54 | M | V, R, C | 1 | 1 | 1 | 2 | 1 | 1 |
| Left Oropharynx | T2N2 | 72 | F | R | - | - | 1 | 2 | 3 | - |
| Mandible and Tongue | T4N2c | 74 | M | R | - | - | 1 | 2 | 3 | 2 |
| Larynx | T4aN0 | 61 | F | R, C | - | - | 1 | 2 | 2 | 2 |
| Right Maxilla | T4N0 | 75 | F | R, C | - | - | 1 | 2 | 2 | 2 |
| Larynx | T4aN1 | 67 | F | R, C | - | - | 1 | 2 | 2 | 2 |
| Tongue | T2N0 | 51 | M | R, C | - | - | 1 | 2 | 2 | 2 |
| Larynx | T4N0 | 62 | M | R | - | - | 1 | 1 | 1 | - |
| Right Mandible | T4N0 | 90 | M | R, C | - | - | 1 | 2 | 2 | 1 |
| Tongue | T2N0 | 34 | M | R | - | - | 1 | 1 | 1 | - |
| Tongue | T4N0 | 53 | M | R | - | - | 1 | 1 | 2 | - |

‘Study element’ indicates which experimental condition samples were allocated to: V - viability study, R - irradiation study and C - the addition of Cisplatin to the irradiation regimen. Information was not available in Parotid case, marked with -. The number of devices set up for each treatment and subsequently used for immunohistochemical analysis is shown.

**References**

1) Gueven, I., Frijters, S., Harting, J., Luding, S., Steeb, H. Granul. Matter, 2017, 19, 1–21.

2) Mayles, WPM., Lake, R., McKenzie, A., Macaulay, E.M., Morgan, HM., Jorda, TJ., Powley, SK. Physics Aspects of Quality Control in Radiotherapy, IPEM Report 81, York, 1999.

3) Allisy-Roberts, P., Williams, J. in Farr’s Physics for Medical Imaging, 2008, pp. 23–47.
